# Asymmetric Dimethylarginine in Chronic Obstructive Pulmonary Disease (ADMA in COPD)

**DOI:** 10.3390/ijms15046062

**Published:** 2014-04-10

**Authors:** Jeremy A. Scott, MyLinh Duongh, Aaron W. Young, Padmaja Subbarao, Gail M. Gauvreau, Hartmut Grasemann

**Affiliations:** 1 Department of Health Sciences, Lakehead University, 955 Oliver Road, Thunder Bay, ON P7B 5E1, Canada; E-Mail: jascott1@lakeheadu.ca; 2 Department of Medicine, McMaster University, 1280 Main Street West, Hamilton, ON L8S 4L8, Canada; E-Mails: duongmy@mcmaster.ca (M.D.); gauvreau@mcmaster.ca (G.M.G.); 3 Department of Physiology and Biophysics, Boston University School of Medicine, 72 East Concord St., Boston, MA 02118, USA; E-Mail: youngaw@bu.edu; 4 Program in Physiology and Experimental Medicine, SickKids Research Institute, and Division of Respiratory Medicine, Department of Pediatrics, Hospital for Sick Children, 555 University Avenue University of Toronto, Toronto, ON M5G 1X8, Canada; E-Mail: padmaja.subbarao@sickkids.ca

**Keywords:** arginine metabolism, nitric oxide, asymmetric dimethylarginine, arginase, pulmonary function, airway obstruction, l-ornithine

## Abstract

l-Arginine metabolism including the nitric oxide (NO) synthase and arginase pathways is important in the maintenance of airways function. We have previously reported that accumulation of asymmetric dimethylarginine (ADMA) in airways, resulting in changes in l-arginine metabolism, contributes to airways obstruction in asthma and cystic fibrosis. Herein, we assessed l-arginine metabolism in airways of patients with chronic obstructive pulmonary disease (COPD). Lung function testing, measurement of fractional exhaled NO (FeNO) and sputum NO metabolites, as well as quantification of l-arginine metabolites (l-arginine, l-ornithine, l-citrulline, ADMA and symmetric dimethylarginine) using liquid chromatography-mass spectrometry (LC-MS) were performed. Concentrations of l-ornithine, the product of arginase activity, correlated directly with l-arginine and ADMA sputum concentrations. FeNO correlated directly with pre- and post-bronchodilator forced expiratory volume in one second (FEV_1_). Sputum arginase activity correlated inversely with total NO metabolite (NO*_x_*) and nitrite concentrations in sputum, and with pre- and post-bronchodilator FEV_1_. These findings suggest that ADMA in COPD airways results in a functionally relevant shift of l-arginine breakdown by the NO synthases towards the arginase pathway, which contributes to airway obstruction in these patients.

## 1. Introduction

**Figure 1.**
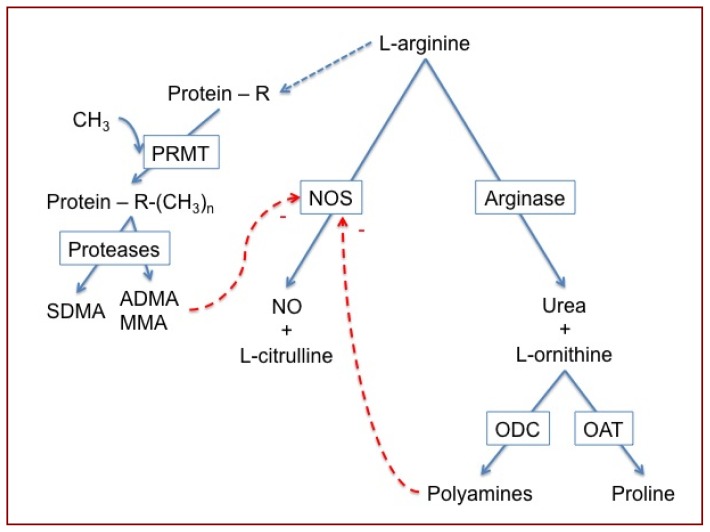
Schema of l-arginine metabolism via the NO synthase (NOS) and arginase pathways to nitric oxide (NO) and l-citrulline, and urea and l-ornithine, respectively. l-Ornithine is metabolized by ornithine decarboxylase (ODC) to polyamines, which can block NOS activity, or by ornithine aminotransferase (OAT), which provides proline, a precursor of collagen biosynthesis. l-Arginine is also incorporated into proteins, where it can be mono- or di-methylated by protein arginine methyltransferases (PRMT), allowing the liberation of monomethyl arginine (MMA) and asymmetric dimethylarginine (ADMA) during proteolysis, which act as endogenous inhibitors of NOS, as well as symmetric dimethylarginine (SDMA), which competes with l-arginine for uptake by the cationic amino acid transporter-2 [1].

l-Arginine metabolism plays an important role in the maintenance of airways tone and function by production of nitric oxide (NO) and l-citrulline, via the NO synthase (NOS) pathway, as well as l-ornithine and the polyamines, via arginase and ornithine decarboxylase, respectively (Figure 1) [1,2]. Dysregulation of the competing enzymes has been shown to contribute to airway obstruction in asthma and in patients with cystic fibrosis [3–5]. Sputum arginase activity is increased in both diseases [2,6–9] and leads to decreased availability of l-arginine substrate for NOS, and subsequently to relative airway NO deficiency. NO is necessary for airway smooth muscle relaxation, and NO deficiency results in airways hyperreactivity [1,2,10,11]. Of the three NOS isozymes, the neuronal and endothelial isoforms are constitutively expressed in airways and contribute to the maintenance of airways smooth muscle tone [12]. The third isoform, the inducible NOS (NOS2) is upregulated in airways inflammation (*i.e.*, airway epithelial cells isolated from patients with asthma [1,13,14]. Thus, the balance between the NOS isozymes and arginases contributes to the tight regulation of airways tone in health and disease.

Recent evidence suggests that arginase controls NOS activity in airways not only through limitation of l-arginine bioavailability, but also by producing l-ornithine, the precursor of polyamine biosynthesis [15,16]. The polyamine spermine, which acts as an inhibitor of NOS, is increased in asthma and CF airways [15,16]. We recently demonstrated in the mouse that nebulization of spermine into the airways led to airways hyperreponsiveness to methacholine, which was mediated via a decrease in airway NO [16]. The shift of l-arginine metabolism toward the arginase pathway has been suggested to contribute to remodeling of asthma airways, due to the production of polyamines [12,16] and of proline, which is a precursor of the deposition of collagen [1,14,17].

NOS activity can also be reduced by the accumulation of endogenous inhibitors such as ADMA, a product of protein degradation that has only recently been recognized as being associated with lung disease [3–5,18,19]. ADMA has long been found to be associated with renal failure [20,21] and cardiovascular disease [22,23]. However, we recently reported increased levels of ADMA in asthma and cystic fibrosis airways [3–5], which likely contributes to NO imbalance and respiratory dysfunction.

Chronic obstructive pulmonary disease (COPD) is clinically characterized by persistent productive cough, mucous plugging, airway obstruction, and progressive airflow limitation on pulmonary function testing [24]. While there has been some evidence for increased arginase expression in airways of smokers with asthma [25] and COPD [26] and altered inducible NO synthase expression in COPD [26], a clear picture of the l-arginine metabolism in the COPD airways remains to be elucidated. Further studies have shown increased arginase expression and/or activity in serum [27], erythrocytes and platelets [28], and bronchoalveolar lavage samples [29] from COPD patients, which have been supported by findings in mouse [29] and guinea pig [30] COPD models. Thus, we quantified the l-arginine metabolites l-arginine, l-ornithine and l-citrulline, as well as surrogate markers of NO synthase activity (FeNO and NO*_x_*) and dysfunction (the endogenous competitive NOS inhibitor ADMA) in sputum samples obtained from patients with COPD and examined their relationship(s) with the outcomes of lung function testing.

## 2. Results and Discussion

### 2.1. Patient Demographics and Lung Function

Lung function testing of COPD patients revealed severe airflow limitation; with a mean pre-bronchodilator FEV_1_ of 1.2 ± 0.1 L (39.6% ± 4.0% predicted) and a significant bronchodilator response (BDR) after inhaled salbutamol of 25.6% ± 4.4% (post-bronchodilator FEV_1_ of 1.5 ± 0.2 L; 49.4% ± 4.8% predicted); thus, severe COPD (Global Initiative for Chronic Obstructive Lung Disease [GOLD] Classification 3) [24]. Patient demographics are presented in Table 1.

### 2.2. Sputum l-Arginine Metabolites and Indices of Arginase and NO Synthase Activity

**Figure 2.**
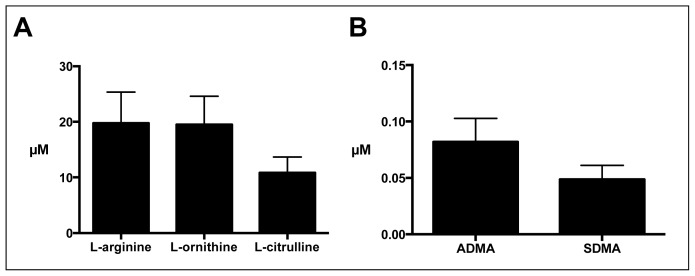
Sputum l-arginine metabolite (l-arginine, l-citrulline, l-ornithine) (**A**); and ADMA and SDMA (**B**), concentrations in COPD patients. Data are expressed as the mean ± SEM.

Sputum concentrations of l-arginine metabolites (*i.e.*, l-arginine, l-citrulline, l-ornithine, ADMA and SDMA) are shown in Figure 2, and sputum NO metabolites (NO*_x_*, nitrite levels) and *in vitro* arginase activity in Table 2. While the l-arginine metabolites were measureable in all COPD samples, the measured concentrations appeared to be lower than in previous studies of CF and asthma sputum samples, but higher than in healthy controls [3,4,31]. While this observation could at least in part be explained by differences in sputum processing, it was interesting to us that the l-arginine/ADMA ratio, an index of NOS impairment, was similar between CF, asthma and COPD sputum samples [6]. Sub-group analyses including smoking state (current/ex-smokers) and inhaled glucocorticosteroid use did not reveal differences, possibly due to the small sample sizes.

### 2.3. Correlations between Sputum Measures of l-Arginine Metabolites and Arginase Activity

**Figure 3.**
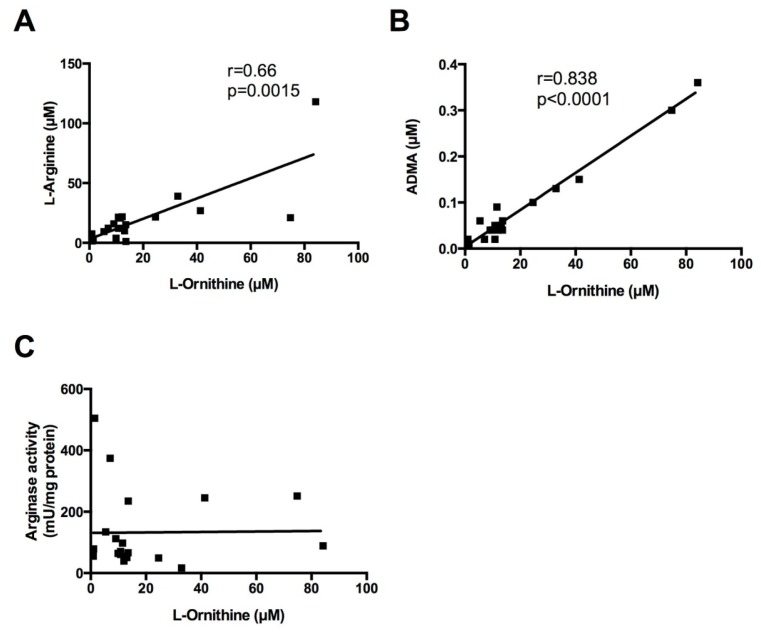
l-Ornithine levels correlate directly with l-arginine (**A**) and ADMA (**B**); but not *in vitro* arginase activity (**C**), in sputum samples obtained from the COPD patients. Spearman’s correlation coefficient and *p*-values are as indicated.

To determine the relationship of the different sputum l-arginine metabolites, we calculated Spearman’s correlation values for each of the parameters. Interestingly, among these measures, l-ornithine correlated directly with l-arginine and ADMA levels in sputum, but not with *in vitro* arginase activity (Figure 3). As l-arginine is the precursor of l-ornithine production along the arginase metabolic pathway, this correlation may be expected. However, the direct and significant correlation between sputum l-ornithine and ADMA levels, which would appear to be due to two independent pathways (*i.e.*, arginase activity and protein turnover, respectively) suggests that higher concentrations of the endogenous NOS inhibitor ADMA result in increased availability of l-arginine for the arginase pathway and subsequently leads to the increased formation of l-ornithine. Such a shift in the NO synthase/arginase balance had previously been suggested to contribute to airways hyperresponsiveness in asthma [2,7], and there has also been evidence from studies in human bronchi that arginase was responsible for exacerbating respiratory sensitivity to spasmogens in COPD patients [26]. There was no correlation of ADMA with FeNO or sputum NO*_x_* concentrations. ADMA did however correlate with l-citrulline concentrations in sputum (Spearman *r* = 0.8651; *p* = 0.0023), which may be explained by the fact that l-citrulline is not only a product of NOS activity but also of ADMA degradation by DDAH [32]. Arginase activity in COPD sputum was 5-fold higher compared to previously reported activities in healthy controls [6]. The observation that sputum arginase activity also did not exhibit significant correlations with ADMA may be explained by the experimental conditions of the *in vitro* assay, which was performed using excess substrate concentrations, and may therefore not be reflective of arginase activity *in vivo*.

### 2.4. Correlations between Sputum l-Arginine Metabolites, Expired NO, Arginase Activity and Lung Function

**Figure 4.**
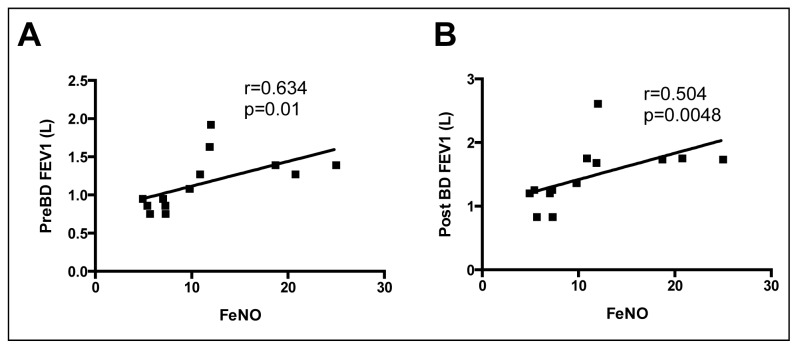
Correlation of the fraction of exhaled nitric oxide (FeNO) with forced expiratory volume in one second (FEV_1_) (**A**) before (pre); and (**B**) after (post), inhaled bronchodilator (BD). Spearman’s correlation coefficient and *p*-values are as indicated.

Based upon our hypothesis that increased ADMA results in reduced NO synthesis, and the fact that NO deficiency has been associated with airflow limitation and airway responsiveness in other respiratory diseases [1,2,10,33,34], it is interesting that the fraction of expired NO (FeNO) in the COPD patients correlated significantly and positively with both pre- and post-bronchodilator FEV_1_ (Figure 4), but not with the bronchodilator induced changes in FEV_1_. All FeNO results were within published normal ranges [35] suggesting that measurements of FeNO may not be helpful to detect abnormalities in the NO metabolism in these patients.

**Figure 5.**
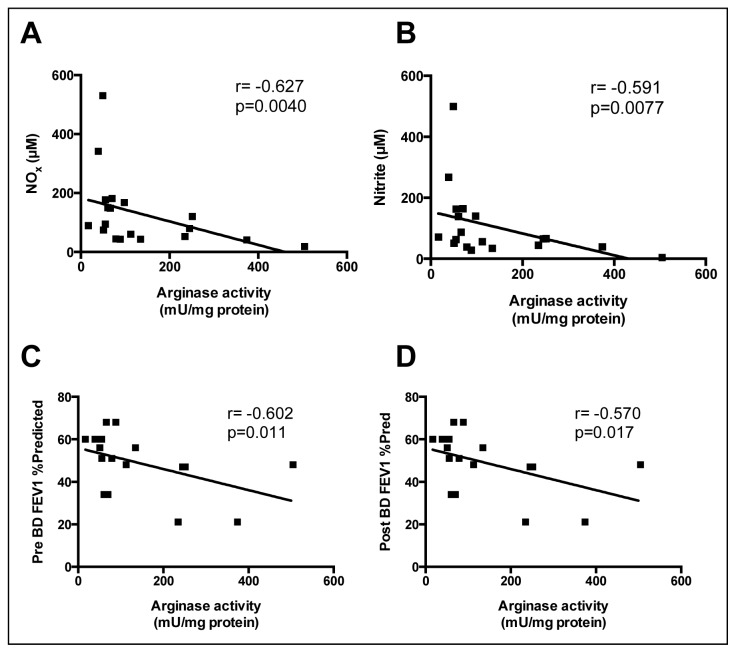
Arginase activity in COPD sputum correlates inversely with sputum NO*_x_* (**A**) and nitrite (**B**) levels, and with both pre (**C**) and post-bronchodilator (**D**) FEV_1_% predicted. Spearman’s correlation coefficient and *p*-values are as indicated.

Further examination of the relationship between arginase activities and levels of NO metabolites (*i.e.*, NO*_x_*, and nitrite) in sputum supported an association between increased arginase activity and impaired production of airway NO. This was further strengthened by the observation that arginase activity correlated significantly and inversely with both pre and post-bronchodilator FEV_1_%predicted (Figure 5).

## 3. Experimental Section

Ten patients with physician-diagnosed COPD [24] were recruited for two independent visits in stable clinical condition. The fraction of exhaled NO (FeNO) [36,37] as well as pulmonary function testing before and after the administration of 400 mcg of salbutamol via metered dose inhaler [38] were measured according to published guidelines of the European Respiratory Society and American Thoracic Society on the first of the two visits, respectively. Sputum specimens were obtained on both visits, mixed with lysis buffer (0.1% Triton X-100 plus added protease inhibitors), homogenized, aliquoted and stored at −80 °C prior to further processing. Sputum specimens were analyzed for l-arginine, l-ornithine and l-citrulline, the endogenous NOS inhibitor ADMA, as well as SDMA using liquid chromatography-tandem mass spectrometry (LC-MS/MS), as previously described [3,4]. Briefly, samples were butylated after deproteinization, separated by high-performance liquid chromatography, and then subjected to mass spectrometry. Concentrations were determined by comparison with standard curves prepared and processed in a similar manner as the sputum samples. Sputum nitrite, NO*_x_*, and *in vitro* arginase activities were measured using spectrophotometric approaches, as described previously [16].

After testing for normality of the distribution of the data, we determined that non-parametric statistical analyses were appropriate. Spearman’s correlations were determined between measures using GraphPad Prism (Version 6.0d, GraphPad Software Inc., LaJolla, CA, USA).

## 4. Conclusions

This is the first assessment of the complex l-arginine metabolism in airways of patients with COPD. Our preliminary analysis demonstrates the presence of the competitive NOS inhibitor ADMA in COPD sputum. The significant correlations between ADMA and l-ornithine, the product of arginase activity, and of arginase activity and NO*_x_*, suggests that accumulation of ADMA in the airways of COPD patients results in a shunt of l-arginine away from NO synthases towards the arginase pathway. The correlation of both arginase activity and FeNO with pre- and post-bronchodilator FEV_1_, further suggests that the observed alterations in the l-arginine metabolism have significant functional consequences for these patients. Increased arginase activity may also contribute to the chronically progressing structural alterations (remodeling) of the COPD airways. It is important to note though, that the alterations in the l-arginine metabolism were not reflected in abnormal FeNO. Further studies are needed to help understand whether sputum analyses could be helpful to assess the effects of therapeutic interventions on the l-arginine metabolism in COPD.

## Figures and Tables

**Table 1.** Patient demographics.

Measure	Mean ± SEM *n*
Age (years) (mean ± SEM)	62.3 ± 2.6
Height (cm) (mean ± SEM)	174.0 ± 1.8
Weight (kg) (mean ± SEM)	76.4 ± 3.0
Gender (m/f)	8/2
Current/Ex smoker	4/6
Pack years	37 ± 5
COPD Diagnosis (years) (mean ± SEM)	7 ± 2.6
Inhaled Glucocorticosteroids (y/n)	6/4
Body Surface Area (m^2^) (mean ± SEM)	1.90 ± 0.04

**Table 2.** Sputum *in vitro* arginase activity, sputum nitrate + nitrate (NO*_x_*) levels and FeNO in COPD patients.

Measure	Mean ± SEM
Arginase activity (mU/mg protein)	132.5 ± 28.4
FeNO (ppb)	17 ± 4
Sputum NO*_x_* (μM)	194 ± 70
